# The role of serum angiopoietin-2 levels in progression and prognosis of lung cancer: A meta-analysis

**DOI:** 10.1097/MD.0000000000008063

**Published:** 2017-09-15

**Authors:** Yuyang Xu, Yingyi Zhang, Zihuai Wang, Nan Chen, Jian Zhou, Lunxu Liu

**Affiliations:** a Department of Thoracic Surgery, West China Hospital; b Western China Collaborative Innovation Center for Early Diagnosis and Multidisciplinary Therapy of Lung Cancer; c West China School of Medicine, Sichuan University, Chengdu, China.

**Keywords:** angiopoietin-2, lung cancer, prognosis, progression

## Abstract

**Background:**

Angiogenesis is an essential process in the development and progression of malignant tumors including lung cancer, in which angiopoietin-2 (Ang-2) plays an important role. The objective of this study was to assess the prognostic value of serum Ang-2 levels in patients with lung cancer.

**Methods:**

A comprehensive systematic electronic search was performed in the Pubmed, Embase, Web of Science, china national knowledge infrastructure, and VIP databases update to October, 2016 (qikan.cqvip.com). Literatures examining the relevance of serum Ang-2 levels to progression and prognosis of lung cancer were eligible for our study. Standardized mean differences (SMD) with 95% confidence interval (95% CI) and a *P* value were applied to compare continuous variables, and hazard ratio (HR) with 95% CI as well as *P* value were applied for prognostic role.

**Results:**

Twenty studies with 1911 patients met the eligibility criteria. Among them, 7 studies with 575 patients with lung cancer assessed the association between expression of serum Ang-2 and prognosis. According to our results, higher levels of serum Ang-2 were associated with the later stage of tumor. Serum Ang-2 levels were significantly lower in stage I than in stage II (SMD: −0.51; 95% CI: −0.75 to −0.27; *P* < .001), in stage II than in stage III (SMD: −0.52; 95% CI: −0.80 to −0.24; *P* < .001), in stage III than in stage IV (SMD: −0.58; 95% CI: −0.93 to −0.23; *P* = .001). In addition, serum Ang-2 levels were higher in patients with lymph node metastasis (SMD: 1.06; 95% CI, 0.57–1.56; *P* < .001). Meanwhile, patients with lung cancer with higher levels of serum Ang-2 were associated with a significant poorer prognosis when compared to those with lower serum Ang-2 levels (HR: 1.64; 95% CI: 1.20–2.25; *P* = .002), and this role was further detected when stratified by ethnicity and histological type.

**Conclusions:**

This systematic review and meta-analysis suggested that serum Ang-2 levels might be a potential predictor for staging, and were associated with prognosis of lung cancer.

## 1 Introduction

Lung cancer is the leading cause of cancer death worldwide.^[1]^ Despite of the widely application of low-dose computed tomography in early detection of lung cancer in recent years,^[2]^ a large proportion of patients present with locally advanced or metastatic disease at the time of diagnosis, which results in a poor prognosis.^[3,4]^ Thus, effective predictors for the progression and prognosis of lung cancer are very important.

Angiogenesis is an essential process in the development and progression of malignant tumors including lung cancer,^[5,6]^ which has been proved in many studies to be controlled by a dynamic balance between vessel regression and growth in which angiopoietins plays a critical role along with vascular endothelial growth factor (VEGF).^[6,7]^ Angiopoietin-1 (Ang-1)^[8,9]^ and angiopoietin-2 (Ang-2)^[10]^ have been identified as ligands for Tie2, which is a receptor tyrosine kinase specifically expressed on endothelial cells (ECs). Ang-1 binds to Tie2, and maintains and stabilizes mature vessels by promoting interaction between ECs and surrounding extracellular matrix. Ang-2 antagonizes the stabilizing ability of Ang-1 by competitively binding to Tie2, which resulting in destabilization of vessels. The vessels destabilized by Ang-2 may undergo angiogenic changes in the presence of angiogenic factors such as VEGF; nevertheless, in the absence of VEGF, these destabilized vessels may undergo regression. Therefore, Ang-2 promotes tumor angiogenesis by priming the vasculature and potentiating the effects of VEGF at the front of active neovascularization.^[7]^

In contrast to predominant expression of Ang-1 and Tie2 in normal lung tissue, Ang-2 is preferentially expressed in lung cancer tissues, which has been implicated as an angiogenic switch in tumorigenesis.^[11]^ Tumors change from an avascular state to an angiogenic state when angiogenic factors for the formation of new blood vessels become dominant over natural angiogenesis inhibitors.^[12,13]^ Subsequently, ECs are activated and lead to Ang-2 expressing intensively, and then the vessels may experience regression. In this situation, the tumor cells might undergo hypoxia, which upregulate VEGF expression to induce tumor angiogenesis conversely. In the presence of VEGF, Ang-2 enables ECs migration, proliferation, and sprouting of new vessels.^[14]^ In addition to this, serum Ang-2 is a useful clinical marker for detecting progression of lung cancer.^[15]^ However, some other studies did report conflicting results that Ang-2 was irrelevant to clinical outcome. Thus, whether serum Ang-2 correlates with prognosis in patients with lung cancer is inconclusive.

Therefore, we performed this systematic review and meta-analysis to assess the clinical significance of serum Ang-2 levels in patients with lung cancer.

## 2 Materials and methods

### 2.1 Ethical approval

This present study did not involve human subjects, so ethical approval was not necessary and informed consent was not required.

### 2.2 Search strategy

We performed a comprehensive systematic electronic search in the Pubmed, Embase, Web of Science, china national knowledge infrastructure (CNKI), and VIP databases update to October 2016 (qikan.cqvip.com). The following terms were used for searching: “Angiopoietin 2” or “Ang-2,” “lung cancer,” “lung carcinoma” or “lung neoplasm,” and all possible combinations. The references reported in the included articles were also considered for completion.

### 2.3 Inclusion and exclusion criteria

The eligible studies in this meta-analysis met the following criteria: investigating the association between serum Ang-2 levels and progression or prognosis or other clinic pathological characteristics related to lung cancer; data were available for further meta-analysis; published in English or Chinese. Articles were excluded if they were published in the form of case reports, letters, reviews, or conference abstracts; insufficient data were provided for further meta-analysis. For the studies containing overlapping patients, we chose the study with the largest number of events to avoid duplication.

### 2.4 Quality assessment

Newcastle-Ottawa Scale (NOS) was performed to assess the quality of each available nonrandomized study.^[16]^ The NOS consists of 3 perspectives with a maximum of 9 stars: a maximum of 4 stars for selection, 2 stars for comparability, and 3 stars for the ascertainment of either the exposure or outcome of interest. Studies labeled with 5 or more NOS scores were considered to be high quality.^[17]^

### 2.5 Data extraction

The eligible articles were reviewed independently by 2 reviewers and any disagreement would be discussed until a consensus was reached. The following essential information were retrieved from the articles including the author, publication year, ethnicity, the source of patients, study design, histological type, stage, age, sex, status of lymph node metastasis, smoking history, and prognosis data. Because the critical value of serum Ang-2 was different in the included studies, we distinguished the concentration of serum Ang-2 into a relative higher level and lower level.

### 2.6 Statistical analysis

To assess the relationship between expression of Ang-2 and prognosis, we measured the hazard ratio (HR) and 95% confidence intervals (95% CIs) as the effective value. If the 2 statistics were given explicitly in the articles, we would use the crude ones, but if not, HR was estimated according to Parmar method.^[18]^ Standardized mean difference (SMD) was applied for continuous variables. The *P* value <.05 was thought to be statistically significant. Chi-square–based *I*^2^ test and Q-statistic test were used to assess the statistical heterogeneity. *I*^2^ < 50% and *P* > .10 suggested acceptable heterogeneity and then a fixed-effect model was applied. Otherwise, significant heterogeneity existed and a random effect model was used. Sensitivity analysis was performed by excluded each study sequentially. All of the calculations were performed by STATA version 12.0 (Stata Corporation, College Station, TX) and Review Manager V.5.3 (The Cochrane Collaboration, Software Update, Oxford, UK).

## 3 Results

### 3.1 Literature selection and study characteristics

**Figure 1:**
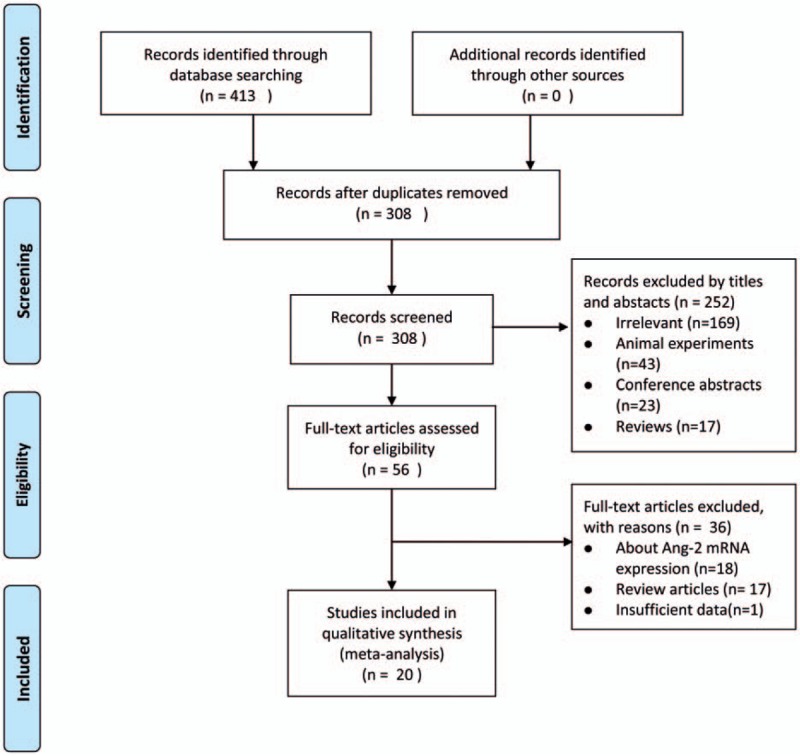
PRISMA flow chart of literature selection. PRISMA = Preferred Reporting Items for Systematic Reviews and Meta-Analyses.

There were 413 citations from the Pubmed, Embase, Web of Science, CNKI, and VIP databases (qikan.cqvip.com). The majority of the researches were eliminated after screening the titles and abstracts. A total of 56 articles were left, and the full texts of which were further evaluated in detail. Finally, 20 studies with 1911 patients in total matched the criteria and were included in the present study.^[15,19–37]^ The flow chart summarizing the systematic literature selection process is shown in Figure 1. Based on the NOS score (Table 1), each study was considered high quality. Seven studies in total assessed the association between expression of serum Ang-2 and prognosis.^[15,21–25,28]^ Among them, 2 studies focused on serum Ang2 levels in small cell lung cancer (SCLC),^[21,28]^ whereas the other 5 studied non–small cell lung cancer (NSCLC).^[15,22–25]^ In addition, 4 studies came from Caucasian institutions,^[21–23,25]^ whereas the other 3 from Asian.^[15,24,28]^ Further information of the included studies are presented in Table 1.

**Table 1:**
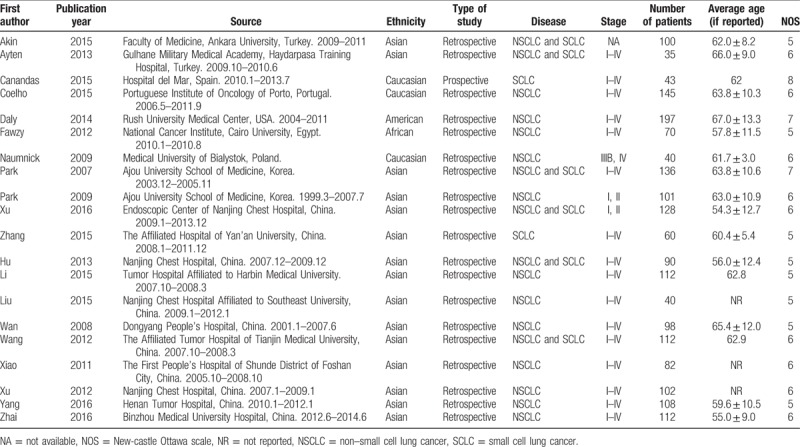
Baseline information of included articles.

### 3.2 Relationship between serum Ang-2 levels and tumor stage as well as other clinical pathological characteristics of lung cancer

**Figure 2:**
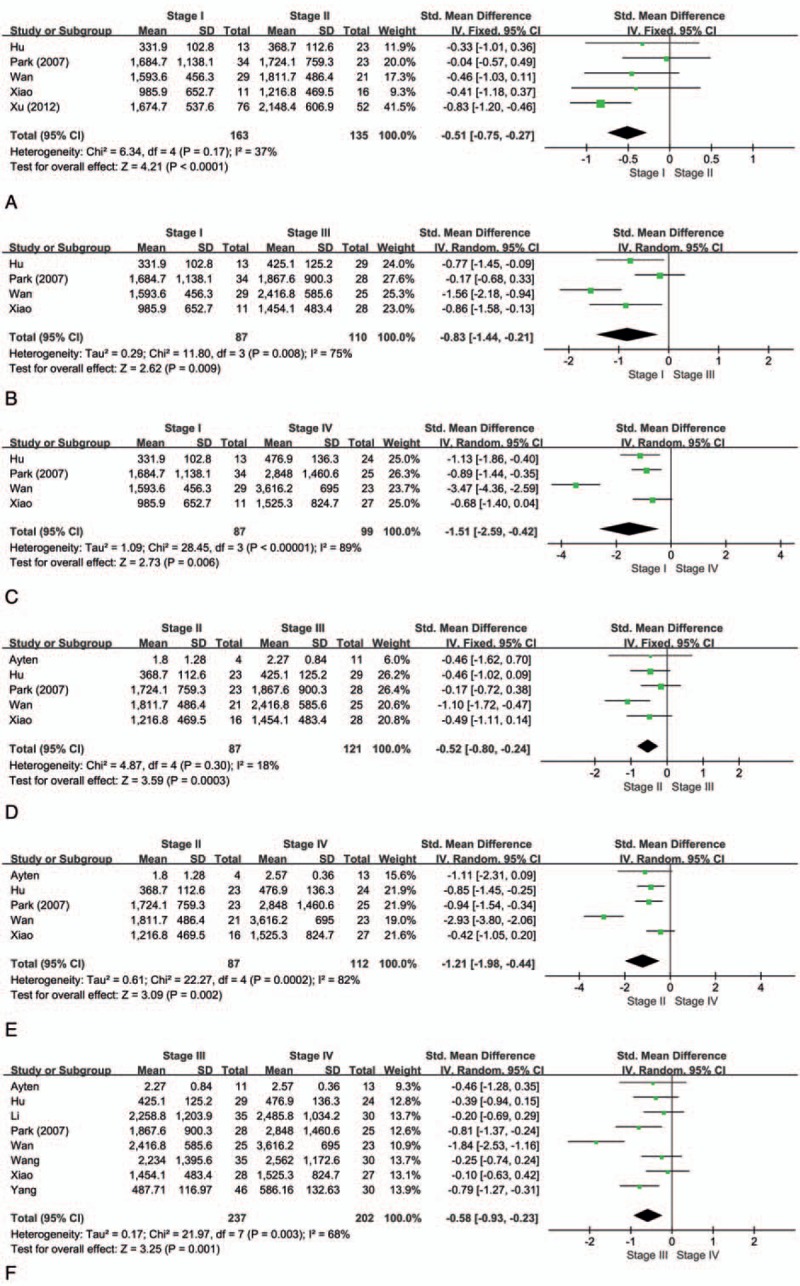
Forest plot of role of SMD and its 95% CI of serum Ang-2 levels between (A) stage I and II, (B) stage I and III, (C) stage I and IV, (D) stage II and III, (E)stage II and IV, and (F) stage III and IV. For each study, the estimate of SMD was plotted with squares, and the size of the square reflects the weight of the study in the meta-analysis. The horizontal line crossing the square represents the 95% CI. The diamond represents the summary SMD and 95% CI. Ang-2 = angiopoietin-2, CI = confidence interval, SMD = standardized mean differences.

**Figure 3:**
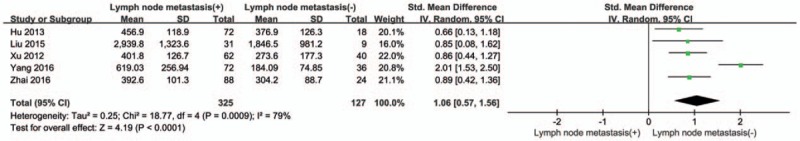
Forest plot of role of SMD and its 95% CI of serum Ang-2 levels between lung cancer patients with and without lymph node metastasis. For each study, the estimate of SMD was plotted with squares, and the size of the square reflects the weight of the study in the meta-analysis. The horizontal line crossing the square represents the 95% CI. The diamond represents the summary SMD and 95% CI. Ang-2 = angiopoietin-2, CI = confidence interval, SMD = standardized mean differences.

There were a total of 9 studies^[15,20,29–35]^ evaluating the correlation of serum Ang-2 levels and stage progression of lung cancer. According to the results of our meta-analysis, higher levels of serum Ang-2 were associated with the later stage of tumor (Table 2). Serum Ang-2 levels were significantly different between stage I and stage II (SMD: −0.51; 95% CI: −0.75 to −0.27; *P* < .001) (Fig. 2A), stage I and stage III (SMD: −0.83; 95% CI: −1.44 to −0.21; *P* = .009) (Fig. 2B), stage I and stage IV (SMD: −1.51; 95% CI: −2.59 to −0.42; *P* = .006) (Fig. 2C), stage II and stage III (SMD: −0.52; 95% CI: −0.80 to −0.24; *P* < .001) (Fig. 2D), stage II and stage IV (SMD: −1.21; 95% CI: −1.98 to −0.44; *P* = .002) (Fig. 2E), as well as stage III and stage IV (SMD: −0.58; 95% CI: −0.93 to −0.23; *P* = .001) (Fig. 2F). Moreover, serum Ang-2 levels were highly associated with lymph node involvement in lung cancer.^[24,29,33,34,36]^ Patients with lymph node metastasis were associated with significant higher serum Ang-2 concentration (SMD: 1.06; 95% CI: 0.57–1.56; *P* < .001) (Fig. 3). However, we did not find any statistical difference between the serum Ang-2 levels and age, sex, smoking history, and histological type (Table 2).

**Table 2:**
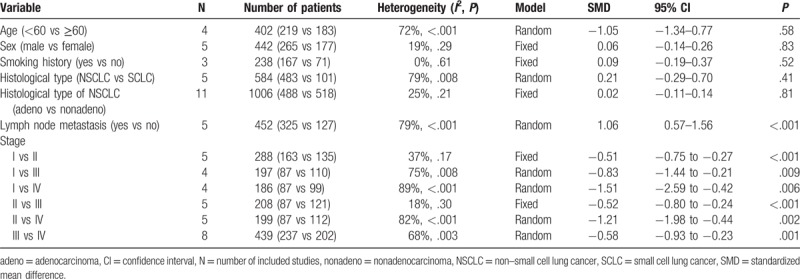
Pooled results of clinical significance of serum angiopoietin-2 levels in lung cancer.

### 3.3 Association between serum Ang-2 levels and overall survival of lung cancer

**Figure 4:**
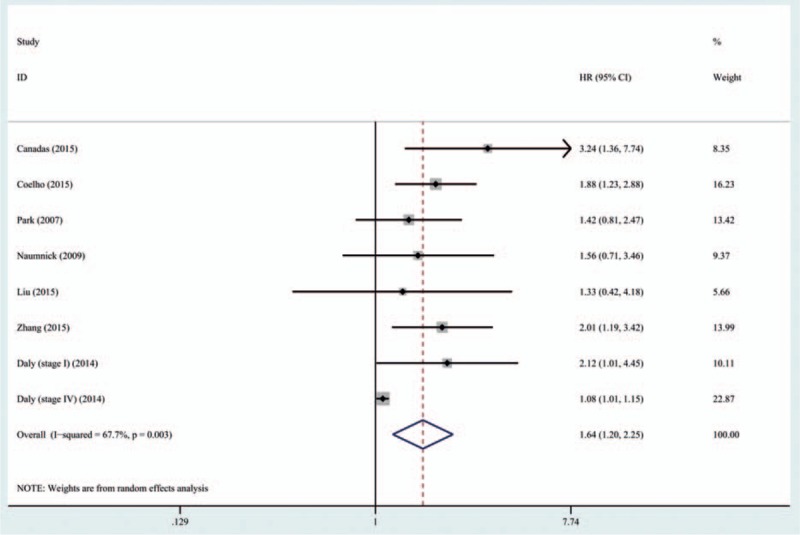
Forest plot of role of serum Ang-2 levels on survival in lung cancer. For each study, the estimate of HR was plotted with squares, and the size of the square reflects the weight of the study in the meta-analysis. The horizontal line crossing the square represents the 95% CI. The diamond represents the summary HR and 95% CI. Ang-2 = angiopoietin-2 , CI = confidence interval, HR = hazard ratio.

In this meta-analysis, a total of 7 studies with 575 patients investigated the association between expression of serum Ang-2 levels and prognosis. High heterogeneity was detected (*P* = .003; *I*^2^ = 67.7%), so the random effects model was applied. Patients with lung cancer with higher levels of serum Ang-2 were associated with a significant poorer prognosis when compared to those with lower serum Ang-2 levels (HR: 1.64; 95% CI: 1.20–2.25; *P* = .002). The results are shown in Figure 4 and Table 3.

**Table 3:**
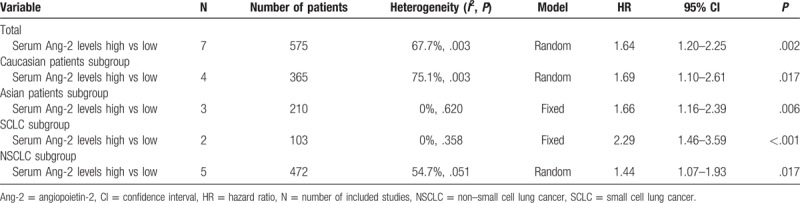
Pooled results of prognostic role of serum angiopoietin-2 levels in lung cancer.

**Figure 5:**
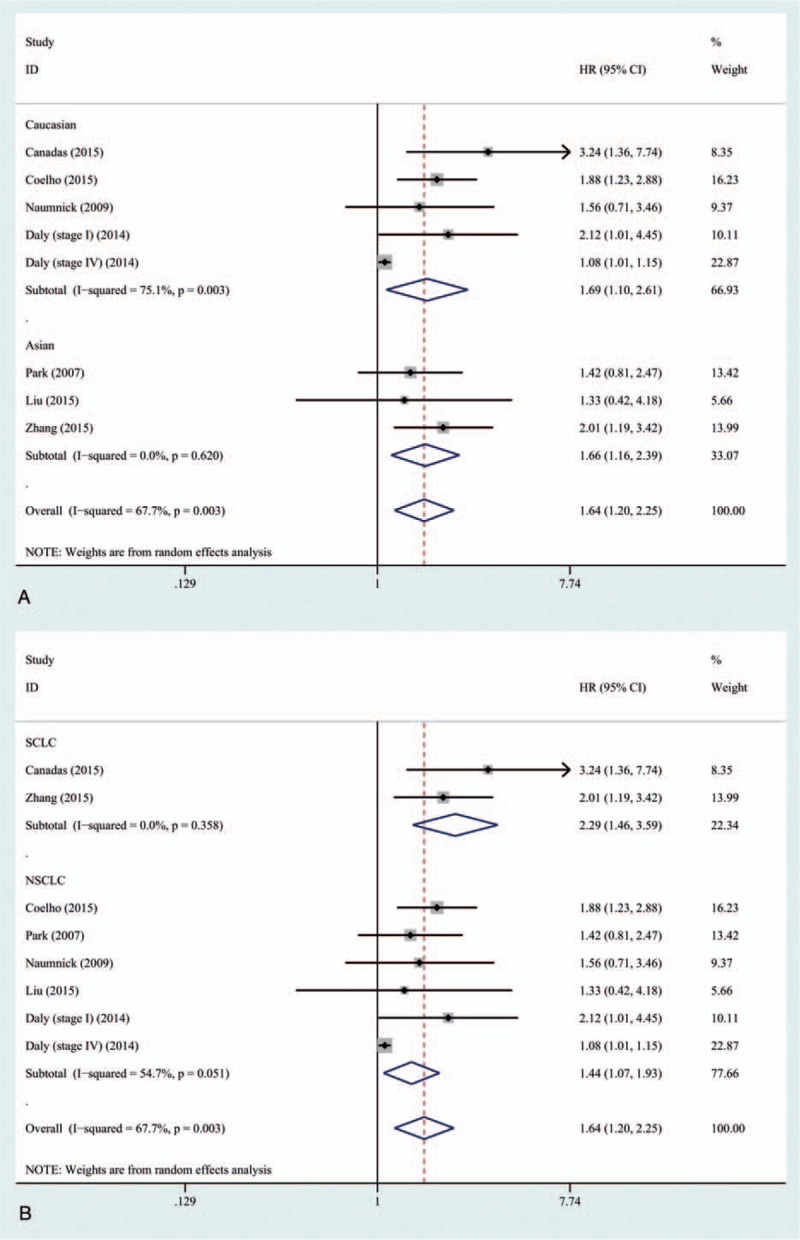
Forest plot of role of serum Ang-2 levels on survival in lung cancer stratified by (A) ethnicity and (B) histological type. For each study, the estimate of HR was plotted with squares, and the size of the square reflects the weight of the study in the meta-analysis. The horizontal line crossing the square represents the 95% CI. The diamond represents the summary HR and 95% CI. Ang-2 = angiopoietin-2 , CI = confidence interval, HR = hazard ratio, NSCLC = non–small cell lung cancer, SCLC = small cell lung cancer.

In subgroup analysis, higher levels of serum Ang-2 indicated poorer outcomes both in Caucasians (HR: 1.69; 95% CI: 1.10–2.61; *P* = .017) and Asians (HR: 1.66; 95% CI, 1.16–2.39; *P* = .006) (Table 3). The heterogeneity of Asian group was acceptable (*P* = .620, *I*^2^ = 0%), whereas it still remained large in Caucasian group (*P* = .003, *I*^2^ = 75.1%) (Fig. 5A). In addition, when stratified by histological type of tumor, higher serum Ang-2 levels were also proved to be a risk factor of poor prognosis in both SCLC (HR: 2.29; 95% CI: 1.46–3.59, *P* < .001) and NSCLC (HR: 1.44; 95% CI: 1.07–1.93, *P* = .017) group (Fig. 5B).

### 3.4 Sensitivity analysis

**Figure 6:**
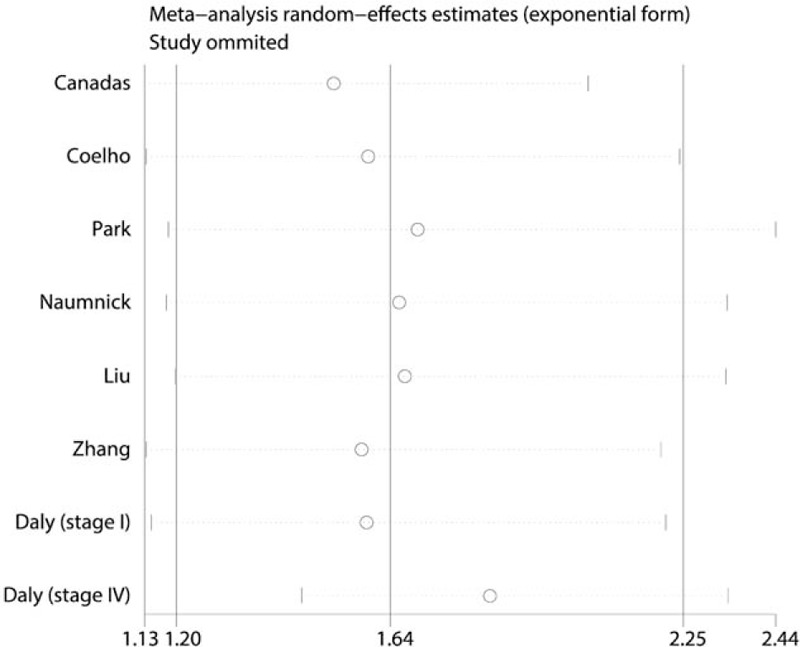
Sensitivity analysis of overall survival. For each study, the circle represents the summary hazard ratio (HR) when excluded this study. The horizontal line crossing the circle represents the 95% confidence interval (CI). The short vertical at the ends of horizontal line represents the lower and upper limit of 95% CI, respectively.

Sensitivity analysis was carried out to evaluate whether the prognosis outcomes were driven by any specific study. No significant changed results were detected by sequential removal of each study based on overall analysis and proved the stability of our findings (Fig. 6).

## 4 Discussion

Lung cancer is one of the most common malignancies, which is the leading cause of cancer-related mortality in the world. In spite of major advances in lung cancer therapeutic strategies, the mortality is still on the rise with unfavorable prognosis and short median overall survival time. Thus, it is essential to identify reliable predictors for lung cancer.

Angiogenesis, which is regulated by a complex balance of proangiogenic and antiangiogenic, plays an important role in carcinogenesis. The degree of angiogenesis was found to be in correlation with the prognosis of patients with lung cancer.^[38]^ Although several preclinical studies addressed some molecules with antiangiogenic activity, the majority of them failed to display efficacy in clinical trials and were abandoned.^[39]^ In recent years, Ang-2 was reported to be a regulator during tumor progression^[40]^ and found to be associated with poor prognosis.^[41]^ Ang-2 expression along with the presence of angiogenic changes in the presence of angiogenic factors such as VEGF at the tumor periphery results in destabilization of vessels, and is associated with promoting further growth of tumor. However, only a few researches documented the correlation between serum Ang-2 and progression and prognosis of lung cancer. A recent meta-analysis from Xuan et al^[42]^ suggested that Ang-2 levels, including in serum, cancer tissues, and mRNA expression, were significantly associated with poor prognosis for patients with NSCLC. Instead, in our current meta-analysis, we reviewed 19 studies and focused on the effect of serum Ang-2 concentration on the role of progression and prognosis in both NSCLC and SCLC.

A linkage between the degree of angiogenesis and metastasis was reported in a variety of malignant tumors, including lung cancer.^[38,43]^ In our meta-analysis, we found the result that for patients with lung cancer, serum Ang-2 levels increased along with the tumor stages. Especially, the result that patients in stage IV had the highest serum Ang-2 levels could corroborate the critical role of Ang-2 in tumor development and metastasis. Meanwhile, significantly higher levels of serum Ang-2 in patients with lung cancer with lymph node metastasis might also reveal Ang-2 to be a potential biomarker in staging. In addition, some previously published studies suggested that the expression of Ang-2 in lung cancer tissues was higher than that in the normal lung tissues.^[11,44]^ These findings aforesaid could plausibly provide theoretical support for that Ang-2 was essential to angiogenesis during tumor progression. Although lung cancer is clinically staged by using whole body imaging currently, patients present with locally advanced or metastatic disease at the time of diagnosis, which also makes it difficult in evaluation of early lung cancer. Thus, determination of serum Ang-2 levels could provide additional information in predicting lung cancer staging, which is noninvasive, convenient, less expensive, and the samples are highly available. Moreover, as current guidelines are to refer patients for imaging examinations only after they have presented with symptoms such as a chronic cough, at which point a typical NSCLC patient generally has fairly advanced and nonresectable tumor. Routine screening of serum Ang-2 levels would be highly advantageous in this group.

For patients with lung cancer, although some evidences have suggested expression of Ang-2 in tumor tissue indicated poor prognosis,^[45–47]^ only a few studies evaluated the levels of Ang-2 in circulation. In 2007, Park et al^[15]^ first reported that in NSCLC, the low Ang-2 group had a better overall survival compared to the high Ang-2 group. Since then, more and more researchers paid attention to the prognostic implications of serum Ang-2 levels in NSCLC. However, there was still no unified conclusion up to now.^[15,19,22–25]^ According to the outcomes of our present meta-analysis, serum Ang-2 levels might be a significantly predictor in lung cancer, and this role extend to both NSCLC and SCLC group. Interestingly, the HR was higher in SCLC group than in NSCLC group. As SCLC typically presents as a more aggressive type of lung cancer, this result might suggest that serum Ang-2 might work as a potential driver of disease aggression. Furthermore, as reported by Zhang et al,^[28]^ “partial response” occurred more frequently among the SCLC patients with the lower serum Ang-2 levels while patients with higher levels were prone to “progressive disease” during combination chemotherapy. This result might interpret the better prognosis in the patients with the lower serum Ang-2 levels compared to the patients with the higher serum Ang-2 levels indirectly.

Nevertheless, we must acknowledge several limitations of our studies. First, the majority of the articles included in our study were retrospective studies (n = 19). The period of follow-up was different among these studies and some follow-up data were derived from description of patient, which probably led to information bias. Second, the inclusion and exclusion criteria differed among studies, so that significant large heterogeneity was detected in statistical outcomes. A possible reason might be the inclusion of patients in stage IV of Daly et al's study,^[23]^ among which the differences of serum Ang-2 levels were not noticeable. Third, since the critical value of serum Ang-2 was different in the included studies, we distinguished the concentration of serum Ang-2 into a relative higher level and lower level. In the future, large-scale randomized studies are still needed to prove the prognostic role of serum Ang-2 levels with an accurate critical value in lung cancer.

In conclusion, this present systematic review and meta-analysis could provide evidence that serum Ang-2 levels might serve as a potential predictor for staging of lung cancer and was significantly associated with prognosis of patients with lung cancer.
